# Enhanced Reactivation of Remapping Place Cells during Aversive Learning

**DOI:** 10.1523/JNEUROSCI.1450-22.2022

**Published:** 2023-03-22

**Authors:** Jake Ormond, Simon A. Serka, Joshua P. Johansen

**Affiliations:** Laboratory for Neural Circuitry of Memory, RIKEN Center for Brain Science, Saitama 351-0198, Japan

## Abstract

Study of the hippocampal place cell system has greatly enhanced our understanding of memory encoding for distinct places, but how episodic memories for distinct experiences occurring within familiar environments are encoded is less clear. We developed a spatial decision-making task in which male rats learned to navigate a multiarm maze to a goal location for food reward while avoiding maze arms in which aversive stimuli were delivered. Task learning induced partial remapping in CA1 place cells, allowing us to identify both remapping and stable cell populations. Remapping cells were recruited into sharp-wave ripples and associated replay events to a greater extent than stable cells, despite having similar firing rates during navigation of the maze. Our results suggest that recruitment into replay events may be a mechanism to incorporate new contextual information into a previously formed and stabilized spatial representation.

**SIGNIFICANCE STATEMENT** Hippocampal place cells provide a map of space that animals use to navigate. This map can change to reflect changes in the physical properties of the environment in which the animal finds itself, and also in response to nonphysical contextual changes, such as changes in the valence of specific locations within that environment. We show here that cells which change their spatial tuning after a change in context are preferentially recruited into sharp-wave ripple-associated replay events compared with stable nonremapping cells. Thus, our data lend strong support to the hypothesis that replay is a mechanism for the storage of new spatial maps.

## Introduction

Episodic memories include details about salient experiences from our past as well as information about the location in which these events occurred (Tulving, 1972). The hippocampus plays a central role in episodic memory and spatial decision-making (Pfeiffer and Foster, 2013, 2015; Wikenheiser and Redish, 2015; Yu and Frank, 2015), and this is thought to rely in part on its ability to create and store unique representations or maps of different spatial and nonspatial contexts (O'Keefe and Nadel, 1978; Eichenbaum, 2017). Alteration of the spatial representation, termed “remapping” (Muller and Kubie, 1987), is also thought to play a role in the encoding of episodic events occurring in familiar environments (Colgin et al., 2008). How this occurs without disrupting the representation of the physical and spatial features of the environment is not clear. Furthermore, the mechanisms through which episodic experiences are integrated into hippocampal representations during learning are not well understood.

Studies in which physical properties of the environment, such as the color or shape of the recording apparatus, are altered have shown that the infield firing rates of existing place fields (PFs) can change in response to changing sensory input without any change in place field location, a process termed “rate remapping” (Leutgeb et al., 2005). Further support for this concept has been found in studies in which changes in behavioral contingencies, such as turning direction on a plus or T-maze, also produce rate remapping (Frank et al., 2000; Wood et al., 2000; Ferbinteanu and Shapiro, 2003). However, studies of remapping during episodic memory encoding using reward or fear learning have reported changes in the place field locations of some, but not all, the recorded place cells (Moita et al., 2003; Smith and Mizumori, 2006; Dupret et al., 2010; Wang et al., 2012; Wu et al., 2017). This partial remapping, meaning remapping in a subset of the place cell population, suggests the possibility of a unique population of place cells that might dynamically encode information relating to events, while leaving a more spatially specific population to stably encode space. But how might episodic memory encoding take place such that it is restricted to one subpopulation, while leaving another unchanged?

One possibility is that encoding cells may be preferentially recruited into sharp-wave ripple (SWR)-associated replay events. Recent work has shown that in CA1, a distinct population of place cells can be identified on the basis of differential recruitment into replay events (Grosmark and Buzsáki, 2016). Further, patterns of activity associated with spatial novelty or reward learning have been shown to recur more frequently in these events (Singer and Frank, 2009; Ambrose et al., 2016). Last, these events have been shown to be important for the stabilization of place fields (Roux et al., 2017) and place cell assemblies in novel environments (van de Ven et al., 2016). However, it is not clear from this previous work whether these memory-encoding mechanisms occur in a specific population of hippocampal neurons.

We set out to explore the existence of an episodic encoding subpopulation of place cells in CA1 and to determine the neural coding mechanisms through which these cells may be selectively integrated into hippocampal networks during learning. Using a novel spatial decision-making task incorporating avoidance of aversive stimuli, we found that remapping cells were preferentially recruited into SWRs and replay events.

## Materials and Methods

### Experimental design and statistical analysis.

A total of four male Long–Evans rats were used. Animals were 6–11 months of age at the time of data collection. Experimental procedures were approved by the Animal Care and Use Committees of the RIKEN Brain Science Institute. Animals were food deprived to ∼85–90% of their baseline weight and trained to nose poke for food reward (sweetened condensed milk diluted with an equal amount of water) on the track. Typically, animals required three to four sessions to reach >100 nose pokes. We then began training the animals to run laps on the full track for food reward. Rats ran an equal proportion of forced-choice and free-choice trials during training. Once animals could run >100 trials, we implanted them with electrode arrays targeting dorsal hippocampus (dHPC), as well as basolateral amygdala (BLA) and lateral entorhinal cortex (LEC; BLA and LEC data are not presented in this article).

Tetrodes were gradually lowered into the CA1 pyramidal cell layer. Rats ran daily sessions on the track during this period. When tetrodes reached the layer, we ran a sequence of two sessions in each animal on consecutive days. In all sessions, the animal left the start/reward zone and ran to a small reward zone where it nosed poke for a small food reward (20 µl of sweetened condensed milk). This triggered a door to close behind it, and another door to open in front of it. It then proceeded to run to a choice point where it could choose one of three arms in free-choice trials, or a single arm because of closing of the other two arms during forced-choice trials, on which to run back to the main reward area where it received a large food reward (100 µl sweetened condensed milk). It was then held in the start/reward zone for 10 s (by closing of the door behind it) before commencement of the next trial.

Both session types were composed of blocks of free-choice trials, forced-choice trials (which always alternated between the three choice arms; e.g., 1-2-3–1-2-3), and combined sequences of forced and free-choice trials. These combined sequences were used to probe the memory/preference of the animal for the “safe” arm while forcing it to occasionally sample the shock arms; these sequences took the form of two free-choice trials followed by a forced-choice trial on one of the shock arms, then two more free-choice trials followed by a forced-choice trial on the other shock arm. To construct maps for remapping analysis (and other analyses restricted to prelearning and postlearning epochs), we recorded prelearning and postlearning baselines (PRE and POST) consisting of four trials on each arm; these could be recorded using 12 forced-choice trials [control (CTRL), both baselines; Learning, prelearning baselines] or four repeats of the free choice–forced choice sequence [using the final four free-choice trials on the safe arm and all eight (4 × 2) forced-choice trials on the shock arms; Learning, prelearning baselines]. In some cases, we recorded additional trials after the postlearning baseline to be used for the awake replay analyses. Each session began with five free-choice trials, allowing us to determine a “least preferred” arm (i.e., the arm chosen least frequently by the animal), which would then be used as the subsequent safe arm in the next session; using the “least preferred” arm allowed us to ensure that subsequent preference for this arm reflected new learning rather than expression of a previously held preference. If there was a tie between 2 arms for the designation of least preferred (i.e., the animal chose one arm five times and the other arms 0 times), we broke the tie by examining the behavioral data of the previous session. A change in contingency was always immediately followed by forced-choice trials to ensure that the animal sampled all arms (pilot experiments indicated that animals were resistant to change their choice behavior away from a previous safe arm to a new safe arm if they were not first forced to sample the new safe arm). Shocks were delivered as bilateral 1 s trains of 1 ms pulses delivered at a frequency of 7 Hz (i.e., a total of seven pulses). Because of differences in shock sensitivity and behavior between animals, the experimenter had to occasionally adjust shock intensity as well as introduce additional forced-choice trials to ensure that the animal learned the new contingencies. Once the contingencies were learned (20 trials above 75% correct choices) and the postlearning baseline was to be recorded, shock intensity had to be reduced to a level where the animal would willingly run through the shock on forced-choice trials but would maintain their preference for the nonshock arm on free-choice trials. Shock intensities ranged from 0.4 to 1.5 mA. The total number of trials run was also determined by how quickly the animal learned the contingencies in the learning sessions, and in all sessions the experimenter had to judge the level of motivation of the animal when deciding to run more trials or end the session. There were no significant differences in the number of trials run between the two session types (two-sample *t* test: *t*_(6)_ = 0.5417, *p* = 0.61).

### Surgical procedures.

Rats were anesthetized with 0.5–1.5% isoflurane. Two craniotomies were made over dHPC (4.2 mm posterior from bregma; ±3 mm lateral from midline). Periorbital shock wires (stainless steel: insulated, 0.003 inch; diameter, 0.055 inch; catalog #791000, A-M Systems) were implanted beneath the skin of each eyelid. The electrode array, containing 26–28 tetrodes targeted to each hippocampus (13–14 tetrodes per hemisphere), was implanted on to the surface of the cortex and electrodes turned 750 µm into the brain. Two bone screws attached to the skull served as ground and reference. Tetrodes (nichrome; Hard Pac coating, 0.25 inch; diameter, 0.0005 inch; item #PF000591, Kanthal) were gold plated to <150 kΩ before implantation. Tetrodes were lowered to dorsal CA1 over 2 weeks, and rats continued to run daily training sessions on the track. Once tetrodes were in the CA1 cell body layer, data collection commenced. Data were acquired using a Neuralynx Digital Lynx acquisition system. Amimals were tracked using custom LEDs imaged by an overhead camera at a frame rate of 30 frames/s.

### Spike sorting.

Spike sorting was performed manually with MClust (A. David Redish, University of Minnesota, Minneapolis, MN) using two-dimensional projections of waveform amplitudes and energies, and autocorrelation and cross-correlation functions as additional separation tools and separation criteria. Excitatory cells were distinguished from interneurons by spike width and average rate; interneurons were excluded from analysis. L-ratio (median, 0.05), isolation distance (median, 27.0; Schmitzer-Torbert et al., 2005), and percentage of interspike intervals <2 ms (median, 0) were used as metrics to assess cluster quality.

### Histology.

In rats 2 and 3, marking lesions were made using 20 µA of anodal current for 10 s; in animals 1 and 4, no marking lesions were made. Animals were transcardially perfused with PBS followed by 4% paraformaldehyde (PFA), brains were cryoprotected in 30% sucrose/4% PFA, and frozen slices of 20 µm (animals 1 and 4) or 40 µm (animals 2 and 3) were cut using a cryostat and stained using either NeuroTrace 530/615 Red Fluorescent Nissl Stain (Thermo Fisher Scientific) or DAPI (Sigma).

### Analysis.

All data analyses were performed in MATLAB.

### Task performance.

**Figure 1.**
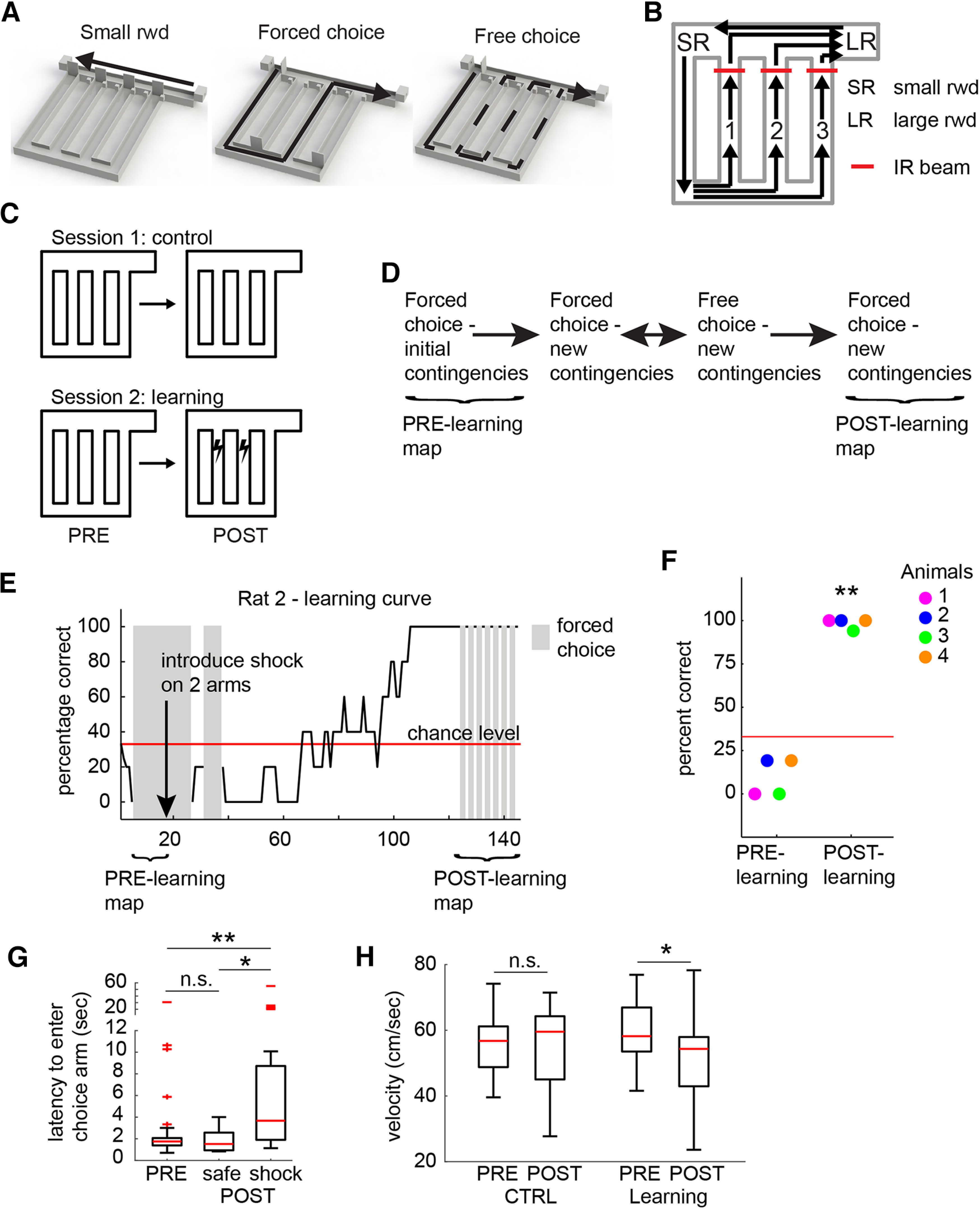
A novel aversive spatial decision-making task. ***A***, On each trial, animals first run to a small reward location (left) and then return to the main large reward location along 1 of the 3 choice arms (middle and right). Sampling of an arm could be forced by closing off the other arms (middle), or could be free choice (right), allowing an assessment of the arm preference of the animal. ***B***, Infrared (IR) beams at the top of the choice arms could be used to trigger eyelid shock. A large food reward was delivered at the end of each trail (top right), while a small food reward was delivered at the top left to motivate the animal to leave the large reward zone. ***C***, Two sessions were run by each of the 4 animals. In the first, animals simply ran along the track, receiving food reward at the reward locations. In the second, after an initial baseline period was recorded, aversive shock was introduced on 2 of the 3 choice arms. ***D***, The sequence of trial types within a session, and their use for the remapping analysis. Note that in a subset of sessions where the animal did not appear to switch its behavior after the first set of “forced-choice-new contingencies” trials, a second set was then run, hence the recursive arrows. ***E***, An example learning curve showing that Rat 2 eventually achieved 100% correct choices. Note that in the final “postlearning” period, forced-choice trials were interleaved with free-choice trials in order for the spatial map on the shock arms to be recorded. ***F***, All animals (4 rats) learned the location of the safe arm (binomial test, *p* < 0.001 for each animal). Note that the experimenter chose the safe arm identity after first running 5 free-choice trials (prelearning) at the outset of the recording session to ensure that animals did not have a bias toward the safe arm before the shock was introduced. ***G***, Latency to enter the shock arm after learning was greater than latency to enter the choice arm before introduction of shock and latency to enter the safe arm after learning. ***H***, Running velocity, averaged within laps, dropped during POST compared with PRE in the learning sessions. *p < 0.05, **p < 0.001, n.s. indicates not significant.

Performance in the task (Fig. 1) was calculated using a moving average (five trials total including the current trial, the two previous. and the two next trials; Fig. 1*E*). In Figure 1*F*, the “PRE” percentage correct was calculated using the first five free-choice trials of the session, and the “POST” percentage correct was calculated using the last 20 free-choice trials of the session.

### Place fields.

**Figure 2.**
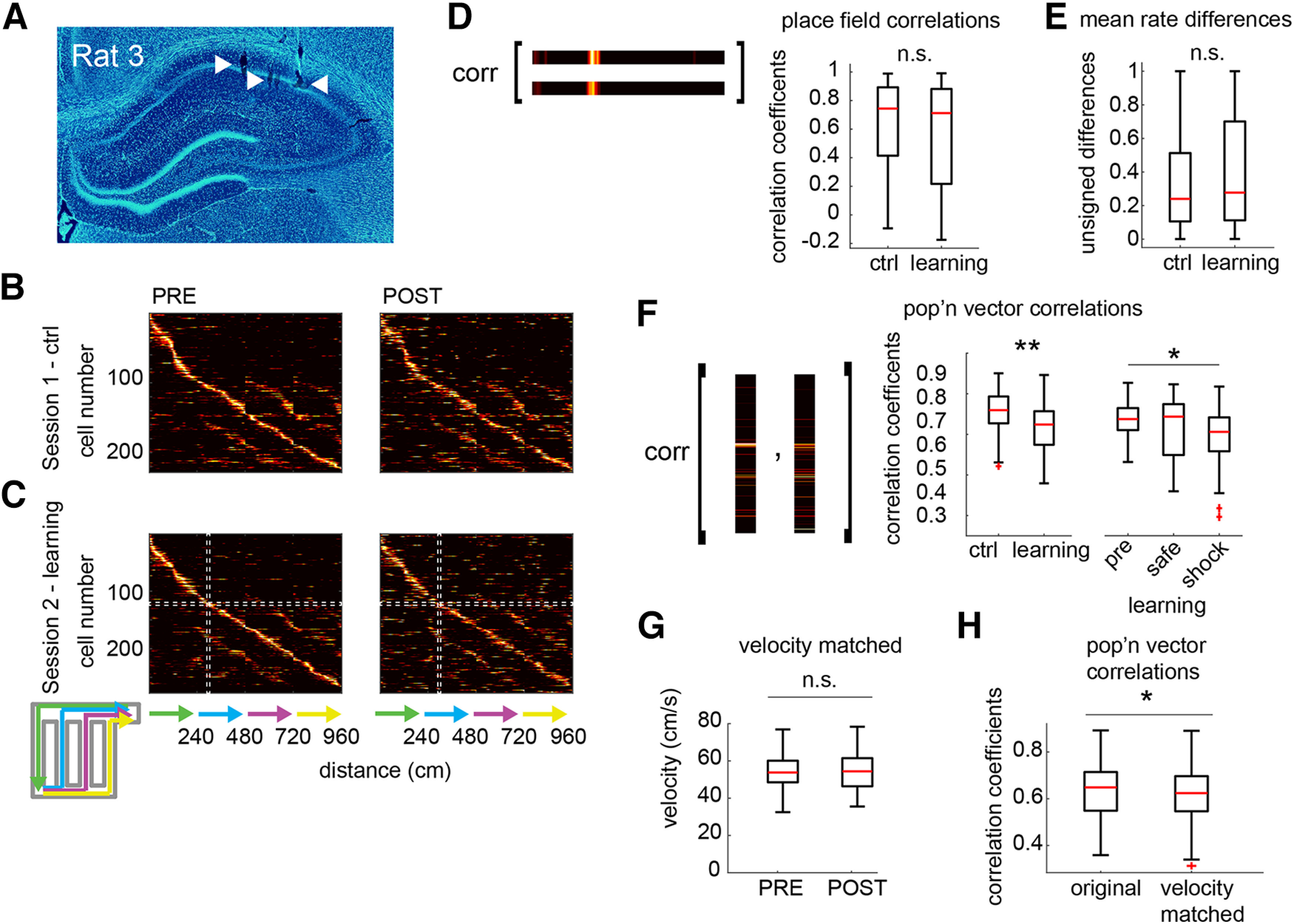
Task learning induces partial remapping. ***A***, Representative histologic section showing positions of tetrode tips in CA1 cell layer. ***B***, Normalized rate maps for all cells recorded during the first (PRE) and final (POST) trials of session 1, in which no shock was introduced. ***C***, As in ***B*** but from Session 2, in which shock was introduced on 2 of the choice arms after the PRE epoch. Schematic at bottom shows how the maze was linearized for place field analysis. White dashed lines outline the rate map of a single cell (horizontal), used in subsequent place field remapping and rate remapping analyses, and a single population vector (vertical), used in subsequent population vector remapping analysis. ***D***, Two representative place cell rate maps from the same unit before and after introduction of the shock in Session 2 (left, outlined by horizontal dashed box in ***C***). PRE-population rate and POST-population rate maps were not significantly less correlated during Session 2 than during control Session 1 (right). ***E***, Rate remapping was not significantly greater in Session 2 than in Session 1. ***F***, Two representative population vectors from the same maze locations before and after introduction of the shock in Session 2 (left, outlined by vertical dashed box in ***C***). PRE-population and POST-population vectors were significantly less correlated during Session 2 than during control Session 1 (right). ***G***, Selecting the slowest laps from the combined set of laps containing both the initial 5 free-choice laps and the PRE epoch, and the fastest laps from the POST epoch abolishes the velocity difference between the PRE and POST epochs. ***H***, Using these velocity-matched laps for the remapping analysis does not reduce the magnitude of the population vector decorrelation, but rather increases it. *p < 0.05, **p < 0.001, n.s. indicates not significant.

Positional data were extracted from the video files, smoothed, and restricted to times when the animal was moving at faster than 5 cm/s for at least 1.5 s (durations of <0.3 s at slower than 5 cm/s within these runs were permitted). The two-dimensional positional data were then linearized (Fig. 2*A*). Each of the four arms (one pre-choice arm, and three choice arms) was divided into 50 spatial bins (4.8 cm/bin), and spikes from individual units were assigned to those bins using linear interpolation. Spike counts were then divided by occupancy at each spatial bin to produce firing rates, and the resulting rate maps were smoothed. A unit with a peak firing rate ≥3 Hz and spatial information ≥0.3 bits/spike was classified as a place cell.

### Remapping.

Population vector (PV) correlations, place field correlations, and mean rate differences were calculated to quantify remapping (Fig. 2). In the population vector analysis, pairs of vectors containing the firing rate of each place cell at a given bin before and after learning were constructed and the Pearson's correlation was calculated. Thus, for each session, there were 200 values of Pearson's *r* (50 bins/arm multiplied by 4 arms) that contributed to subsequent statistical analysis. In the place field correlation analysis, the Pearson's correlation was calculated for pairs of prerate and postrate maps for individual place fields. In the mean rate difference analysis, the mean rate of the PRE rate map was subtracted from the mean rate of the POST rate map, and the absolute of this value was divided by the sum of the two mean rates (thus, a value of 1 indicates 0 firing in either the PRE rate or POST rate epoch, and a value of 0 indicates identical mean rates in the two epochs).

### Identifying remapping cells.

**Figure 3.**
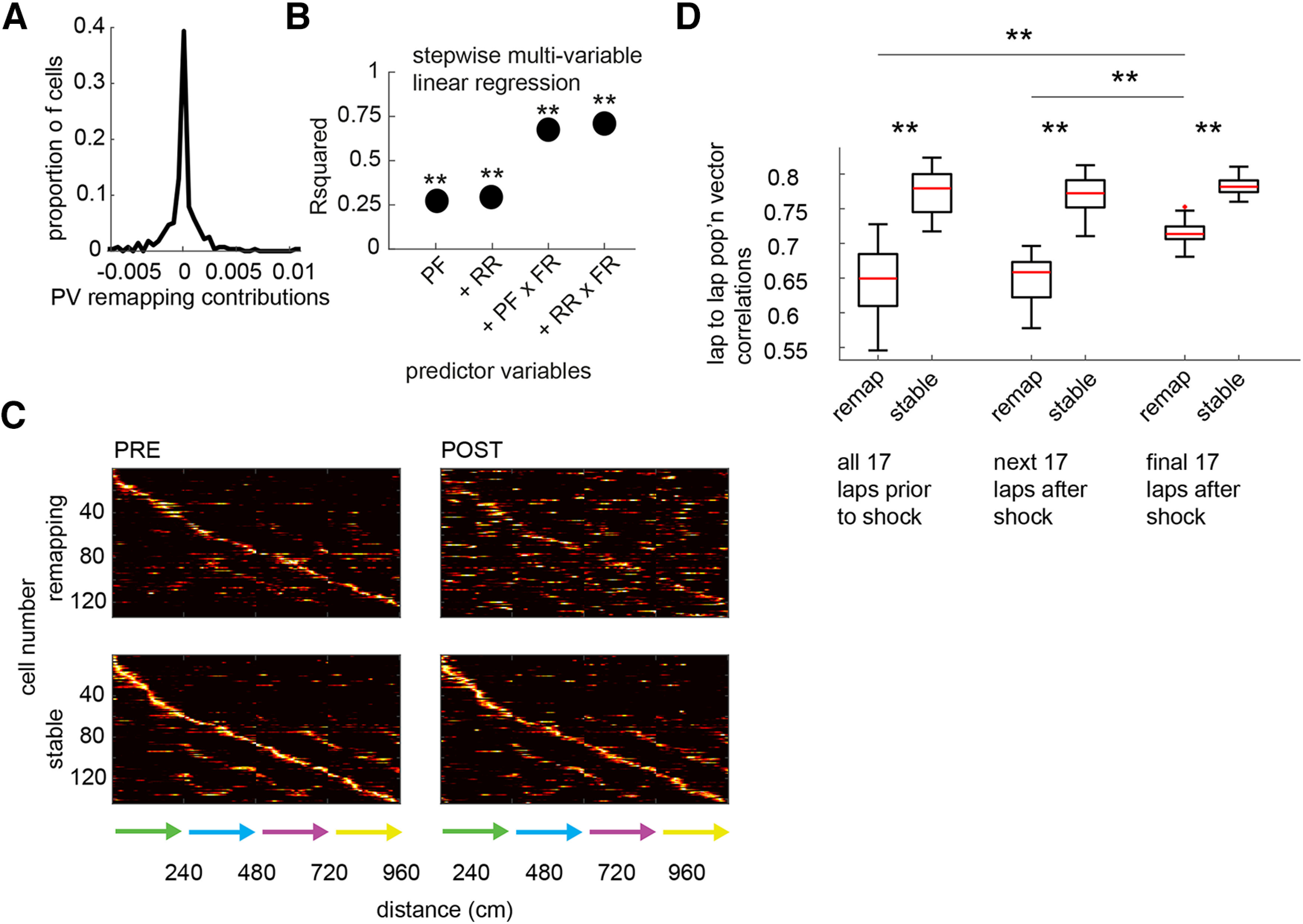
Splitting the place cell population into remapping and stable subpopulations. ***A***, The contribution of each cell to PV decorrelation during Session 2 (Fig. 2*F*) was calculated. No clear bimodality was observed, suggesting a continuous distribution of place cells with highly remapping and stable cells at the extremes of the distribution. ***B***, Stepwise multivariable linear regression showed that a cell's PV remapping contribution was significantly correlated with its PF remapping (PF), its rate remapping (RR), and the products of these remapping values with its maximal firing rate (FR; *t* tests, all *p* < 0.001). ***C***, Session 2 cells were split into remapping and stable groups according to whether they had positive or negative PV remapping contributions. Note that both classes of cells had place fields distributed across the full extent of the maze. ***D***, Remapping cells were less stable than stable cells at all time points. However, they were significantly more stable at the final time point when the contingencies were well learned. **p < 0.001

Rate maps from each recorded cell were combined to create prerate and postrate maps of the entire population (Fig. 3*A*). For each cell, the contribution of each individual rate map to decorrelation of the PVs (i.e., remapping) was calculated as follows: first, the population of PV correlations between all prelearning and postlearning rate maps were calculated, which was then used to calculate a mean PV correlation value; second, the PV correlations were recalculated *N* times, where *N* refers to the number of place cells, each time omitting the rate map of the *n*th cell, and these were used to generate *N* mean PV correlation values; and third, the mean value generated using all rate maps was subtracted from the mean values generated from the partial sets of rate maps to generate a remapping contribution value for each held-out cell (e.g., a place cell that remaps will generate a larger score because the mean PV correlation will be higher when that cell is omitted than when it is included).

### Stepwise multivariable linear regression.

All data (i.e., remapping contribution, place field correlations, mean rate difference, and maximum firing rates) were *z* scored (Fig. 3*B*). We then used the built-in MATLAB function *fitglm* with four combinations of place field correlations, mean rate difference, and maximum firing rates as the predictor variables (combination 1, place field correlations; combination 2, place field correlations and mean rate differences; combination 3, place field correlations, mean rate differences, and place field correlations multiplied by maximal firing rates; combination 4, place field correlations, mean rate differences, place field correlations multiplied by maximal firing rates, and mean rate differences multiplied by maximal firing rates) and remapping contribution as the response variable to produce *r*^2^ and *p*-values for each predictor combination.

### Burst index.

The burst index (see Fig. 5*A*) was calculated as the number of spikes fired in bursts, which were defined as three or more consecutive spikes fired with interspike intervals <8 ms, divided by the total number of spikes (Mizuseki and Buzsáki, 2013).

### Replay analysis.

We defined (see Fig. 7) candidate awake replay events as population events with durations of at least 100 ms occurring when the velocity of an animal was <5 cm/s and characterized by a peak elevation of the multiunit firing rate of at least 3 SDs above the mean, a minimum firing rate of no less than half the mean, and accompanied by a significant increase in local field potential power in the ripple frequency. To calculate the frequency of an individual ripple (Fig. 6*C*), we plotted the spectrogram of the ripple and took the frequency as the location along the frequency axis of any local maximum (i.e., a bump); if no local maximum was found, the ripple was excluded from analysis (42 of 88 before the contingency change; 531 of 912 after obtaining clear maxima).

To analyze ripple-centered firing, we calculated the mean firing rate in a 100 ms window centered on the time when the peak power between 150 and 300 Hz occurs. To calculate the average ripple-centered firing rate (see Fig. 7*C*), we calculated the collective firing rates of the remapping and stable populations within each ripple event, and divided each by the total number cells in that population to give the firing rate per cell (see Fig. 7*D* for how the ripple-centered firing rate for each cell averaged across all ripples was calculated; these values were then used in the correlational analysis seen in Fig. 7*E*).

The patterns of spiking during population events were decoded using a Bayesian decoding algorithm (Zhang et al., 1998; Davidson et al., 2009). Because the spatial representation partially remapped during the session, we updated the rate maps used as templates for the decoding on every lap, and for decoding of a given event, we used the rate map corresponding to the lap during which it had occurred. The template was created identically to the rate maps shown in Figure 2; that is, by concatenating the paths along the prechoice portion of the maze with the paths along each choice arm (Fig. 2*C*, bottom schematic). Each event was subdivided into overlapping 20 ms windows (step size, 5 ms). The probability of spiking activity in a given window of the event corresponding to a position on the track was given by the following:

$$\text{Pr}(\text{pos|spikes})=(\overset{n}{\underset{i=1}{\prod }}f_{i}{\left(\text{pos}\right)}^{\begin{array}{c} sp_{i} \end{array}})e^{-\tau \overset{n}{\underset{i=1}{\sum }}f_{i}(\text{pos})},$$

where *f_i_*(pos) is the value of firing rate by position vector of the *i*th unit at position pos in the template, *sp_i_* is the number of spikes fired by the *i*th unit, τ is the time window duration (20 ms), and *n* is the total number of cells. Posterior probabilities were normalized as follows:

$$\text{Pr}(\text{pos|spikes})=\frac{\text{Pr}(\text{pos}|\text{spikes})}{\begin{array}{c} \sum_{i=1}^{P_{n}}\text{Pr}({\text{pos}}_{i}|\text{spikes}) \end{array}},$$

where *P_n_* is the total number of positions. Putative events were discarded if fewer than four place cells fired spikes. To verify that the algorithm decoded the position accurately, we decoded the position from spikes fired in 100 ms windows while the animal ran the task (see Fig. 7*A*). The decoded position was taken as the spatial bin with highest posterior probability in a given time window.

To determine the quality of a given replay event, we calculated a replay sequence score according to the methods of Grosmark and Buzsáki (2016). First, the weighted mean was calculated as follows:

$$m\left(\text{pos};\text{Pr}\right)=\overset{M}{\underset{i=1}{\sum }}\overset{N}{\underset{j=1}{\sum }}{\text{ Pr}}_{ij}{\text{pos}}_{j}/\overset{M}{\underset{i=1}{\sum }}\overset{N}{\underset{j=1}{\sum }}{\text{ Pr}}_{ij}.$$

The weighted covariance was calculated as follows:

$$\text{cov}\left(\text{pos,bin;Pr}\right)=\frac{\sum_{i=1}^{M}\sum_{j=1}^{N}{\text{ Pr}}_{ij}({\text{pos}}_{j}-m\left(\text{pos};\text{Pr})\right)/\left({\text{bin}}_{i}-m\left(\text{pos};\text{Pr}\right)\right)}{\sum_{i=1}^{M}\sum_{j=1}^{N}{\text{ Pr}}_{ij}},$$

and the weighted correlation was calculated as follows:

$$r\left(\text{pos,bin;Pr}\right)=\frac{\text{cov}\left(\text{pos,bin;Pr}\right)}{\sqrt{\text{cov}\left(\text{pos,pos;Pr}\right)\text{cov}\left(\text{bin,bin;Pr}\right)}},$$

where Pos*_j_* is the *j*th spatial bin, bin*_i_* is the *i*th time bin in the event, Pr*_ij_* is the Bayesian posterior probability for pos*_j_* and bin*_i_*, *M* is the total number of time bins, and *N* is the total number of spatial bins.

We then decoded the events using 1000 templates in which we circularly translated unsmoothed rate maps by a random number of bins separately for each unit and then smoothed them (place cell shuffle), and 1000 template templates in which we circularly translated the population vectors at each spatial bin separately for each population vector (population vector shuffle). A weighted correlation was then calculated for each shuffled event. Because we used six template maps for decoding, we applied a correction for statistical significance testing; an event was considered statistically significant if its absolute weighted correlation was >99.2% of absolute place cell shuffled event-weighted correlations (i.e., *p* = 0.05/6 = 0.0083) and 99.2% of the population vector shuffled event-weighted correlations (*p* = 0.0083).

To determine the per cell contribution (PCC), we first calculated sequence scores for each significant event as follows:

$$rZ=\text{min}\left(\frac{\left|r(\text{observed}|-|\bar{r\left({\text{null}}_{\text{PF}}\right)}\right|}{\text{SD}(\left|r\left({\text{null}}_{\text{PF}}\right)\right|)},\frac{\left|r(\text{observed}|-|\bar{r\left({\text{null}}_{\text{PV}}\right)}\right|}{\text{SD}(\left|r\left({\text{null}}_{\text{PV}}\right)\right|)}\right),$$

where *r*(null_PF_) and *r*(null_PV_) denote the place field-shuffled and population vector-shuffled distribution of weighted correlations, respectively, and SD their standard deviations. Then, for each event, the contribution of each participating cell was determined by calculating the weighted correlation for that event using a template in which only the firing rate by position vector of that unit was taken from the shuffled rate maps; if *rZ* = *rZ*_PF_, then the place field-shuffled maps were used, otherwise the population vector-shuffled maps were used. This was repeated 1000 times (i.e., using a different shuffled vector on each iteration), and a sequence score corresponding to the shuffled unit and the type of shuffle (i.e., place field or population vector) was calculated as follows:

$$rZ_{\text{shuffled}}=\frac{\left|\bar{r(\text{shuffled})}|-|\bar{r\left(\text{null}\right)}\right|}{\text{SD}(\left|r\left(\text{null}\right)\right|)},$$

and then:

$$\text{PCC}=\left[rZ-rZ_{\text{shuffled}}\right]\text{ * }n\mathrm{CellsParticipating}\text{.}$$

For each cell, PCC scores were averaged across all events within which it participated (see Fig. 5*C*). In a separate analysis, the likelihood of a cell participating in replay events was determined by dividing the number of significant events in which the cell fired at least 1 spike by the total number of significant events (see Fig. 5*D*). As seen in Figure 5, *E* and *F*, for each ripple we calculated the proportion of remapping and the number of stable cells active within it, as well as the number of spikes fired by each population divided by the number of cells in that population.

Note that replay analysis used all postcontingency change trials, labeled as “POST-all.” The ripple analysis used all precontingency change trials, including the five additional free-choice trials that occurred immediately before the PRE baseline, labeled as “PRE-all,” as well as the same POST-all epoch used in the replay analysis.

### Statistics.

All statistical analyses were performed in MATLAB. Paired *t* tests were used to assess the behavioral data in Figure 1; elsewhere, except where noted, the Wilcoxon rank-sum test was used to assess the significance of the difference between two groups, whereas the Wilcoxon signed-rank test was used to test differences within a group. Summary data were presented as box plots; the bottom and top of the central boxes represent the 25th and 75th percentiles, with the central line representing the median, whiskers extending a maximum of 1.5 * length of the 25th to 75th percentile distance, and any additional data points plotted as outliers.

### Data availability.

All code used in this study is available by request from the corresponding authors.

## Results

### Animals learn to avoid the shock arm when navigating to the food reward

In reward-learning paradigms, remapping occurs mainly around reward locations (Breese et al., 1989; Dupret et al., 2010; Gauthier and Tank, 2018). Thus, any effort to determine what intrinsic properties cause some place cells to remap while others do not is bound to be confounded by the locations of the place fields of the cells. To circumvent this limitation, we developed a novel decision-making task incorporating aversive stimuli, which produce spatially distributed remapping of hippocampal place cells (Moita et al., 2004; Wang et al., 2012; Wu et al., 2017). The task requires animals to use spatial knowledge about a stable, familiar environment in conjunction with information about changing episodic experiences occurring within this environment, leading to the creation of powerful episodic memories. Rats (*n* = 4) were trained to run laps on a track arranged such that they first ran to a feeder point for a small food reward, followed by a second short run to a choice point where they could freely choose, or alternatively be forced, to run along one of three choice arms to return to a goal location for a larger food reward (Fig. 1*A*). Rats were implanted with eyelid shock wires, and shock (a 1 s train of 1 ms pulses at 7 Hz, with intensity between 0.4 and 1.5 mA) could be triggered by breaking an infrared beam on the choice arms (Fig. 1*B*). During initial training, rats learned to navigate to the reward location; animals were never exposed to shock during this initial training. The experiment consisted of two sessions. In the first session [Fig. 1*C*, Session 1: control (CTRL)], rats ran a large number of laps in the absence of shock (laps: *n* = 132, 105, 97, and 114 for rats 1–4, respectively). The first and final 12 trials consisted of forced choice, with equal sampling of the three choice arms (i.e., each arm was run four times during both the PRE-learning and POST-learning epochs), and these were used to construct PRE-learning and POST-learning rate maps to assess baseline levels of place field stability. In the second session (Fig. 1*C*, Session 2: Learning), rats first ran five free-choice trials (Fig. 1*F*, PRE-learning) to determine their arm preferences. This was followed by 12 forced-choice trials (PRE), as in session 1, to establish the baseline rate maps. Shock was then introduced on two of the choice arms, making these the “shock” arms and the remaining arm the safe arm (the arm least preferred by the animal was chosen as the safe arm). A variable number of forced-choice trials was then run to introduce the new contingencies to the animal (see Materials and Methods), followed by free-choice trials allowing the experimenter to assess the preference of the animal for the safe arm. Once the animal reached 100% correct choices, we interleaved free-choice trials with forced-choice trials on the shock arms to record postlearning rate maps for all three choice arms (Fig. 1*E*, POST-learning); all but one of the animals (rat 2, one error) made 100% correct choices during this postlearning epoch (choices significantly nonrandom; binomial test, *p* < 0.001 for each animal; Fig. 1*F*). The latency with which animals entered the shock arms after learning was significantly greater than both the latency before introduction of the shock and the latency to enter the safe arm after learning (Kruskal–Wallis test followed by *post hoc* Dunn–Sidak test, *H*_(95)_ = 17.8, *p* < 0.001; Fig. 1*G*); in subsequent remapping analysis, we velocity thresholded our data to remove times when the animal hesitated to enter a choice arm (see Materials and Methods). Further, there was a significant reduction in running velocity from PRE to POST that did not occur in CTRL sessions (Wilcoxon rank-sum test; CTRL: *z* = −0.81, *p* = 0.42; Learning: *z* = 3.3126, *p* = 0.0017; Fig. 1*H*).

### A subset of CA1 place cells remap after aversive learning

Once dorsal CA1 tetrodes reached their target (Fig. 2*A*), experiments commenced. We recorded 228 place cells during the CTRL session (Fig. 2*B*) and 277 place cells during the Learning session (Fig. 2*C*; place cells defined as having a minimum spatial information score of 0.3 bits/spike, and a minimum peak firing rate of 3 Hz). The paths of the animal along the track were linearized and concatenated (Fig. 2*C*, bottom) to allow for the remapping analysis. We assessed remapping using the following three commonly used measures: (1) place field correlations; (2) mean rate differences; and (3) population vector correlations. Surprisingly, place field correlations for cells recorded during the Learning session were not significantly lower than for those recorded during the CTRL session (Wilcoxon rank-sum test, *n* = 228 and 277 units, *z* = 1.72, *p* = 0.085; Fig. 2*D*). This result indicated a lack of what is often referred to as “global” remapping, where place field locations change, but left open the possibility of rate remapping, whereby the in-field firing rates of place cells change in an uncoordinated fashion across the population. However, mean rate differences, typically used to assess rate remapping (Leutgeb et al., 2005), were not significantly greater after learning compared with control sessions (Wilcoxon rank-sum test, *z* = −1.43, *p* = 0.154; Fig. 2*E*). Finally, we examined correlations between population vectors corresponding to the firing rates of each cell at individual spatial bins (Fig. 2*F*, left). In contrast to the place field correlations and mean rate differences, population vector correlations after learning were significantly lower than those from the control session, indicating that remapping had indeed occurred [Wilcoxon rank-sum test, *n* = 800 (200 population vectors from each session), *z* = 7.31, *p* < 0.001; Fig. 2*F*, right]. This remapping was not an artifact of lower running velocities during POST as replacing the fastest PRE and slowest POST laps so as to have velocity match the two epochs (Wilcoxon rank-sum, *z* = −0.026, *p* = 0.98; Fig. 2*G*; see Materials and Methods) increased, rather than diminished, the magnitude of remapping (Wilcoxon sign-rank test, *z* = 2.54, *p* = 0.011; Fig. 2*I*).

What can explain the discrepancy between the place field correlations and mean rate differences, on the one hand, and the population vector correlations on the other? Partial remapping, in which only a subset of cells remap, could produce this result if the subset of remapping cells had place fields distributed across all spatial bins, as this would cause each pair of population vectors to be slightly more decorrelated than they otherwise would be. To confirm this, we first transformed the population vector data into scores that could be assigned to individual cells. We did this by performing a subtraction analysis in which population vector correlations were recalculated using population vectors in which a given place cell was omitted. A “remapping contribution” score was then calculated for each place cell as the difference between the population vector correlations using all place cells and the correlations using all but that particular cell. Thus, a cell that contributed to decorrelation received a positive remapping contribution score, and a cell that instead contributed to stability (i.e., the correlations were reduced with that cell omitted) received a negative remapping contribution score (Fig. 3*A*); for each of the four rats, we classified 25, 37, 39, and 32 place cells as remapping, and 21, 38, 26, and 59 as stable, respectively. We performed a stepwise regression to create a generalized linear model to determine which variables best predicted the final remapping contribution score; as variables, we included the place field correlation of each cell, its mean rate difference, as well as its maximal firing rate. The remapping contribution score for a given cell was best modeled as a combination of its place field correlations, its mean rate difference, as well as interactions (i.e., product of terms) between each of these variables and the maximal firing rate of the cell (*t*_(275)_ = −10.5, 4.8, −11.3, 5.7, respectively; all *p* < 0.001; Fig. 3*B*). Thus, a cell whose fields remap but whose firing rate is relatively low will make a smaller contribution to remapping at the population level than a similar cell with a relatively high firing rate.

To compare cells that remapped to cells that did not, we classified all cells with positive remapping contributions as remapping cells, and cells with negative contributions as “stable” cells (*n* = 133 remapping cells, 144 stable cells). Replotting the rate maps for cells classified in this way showed that both remapping and stable cells were distributed across the maze, and that the remapping population was composed both of cells whose place field locations changed and rate-remapping cells whose field locations did not change (Fig. 3*C*). We examined the stability of remapping and stable cells by calculating PV correlations between rate maps constructed from individual consecutive laps. We then calculated the average PV correlation for each pair of consecutive laps and grouped them according to whether the lap occurred before the contingency change, immediately after, or at the end of the session. Remapping cells were less stable than stable cells at all time points (Wilcoxon signed-rank test, *z* = −3.52, *p* < 0.001 at each time point; Fig. 3*D*), including before the contingency change. However, they were significantly more stable at the final time point (Kruskal–Wallis test, *post hoc* Dunn–Sidak; *H*_(47)_ = 25.5, *p* < 0.001; stable cells: *H*_(47)_ = 1.18, *p* = 0.56; Fig. 3*D*). This shows that remapping cells were initially unstable in their representations, but became stabilized over the course of learning.

**Figure 4.**
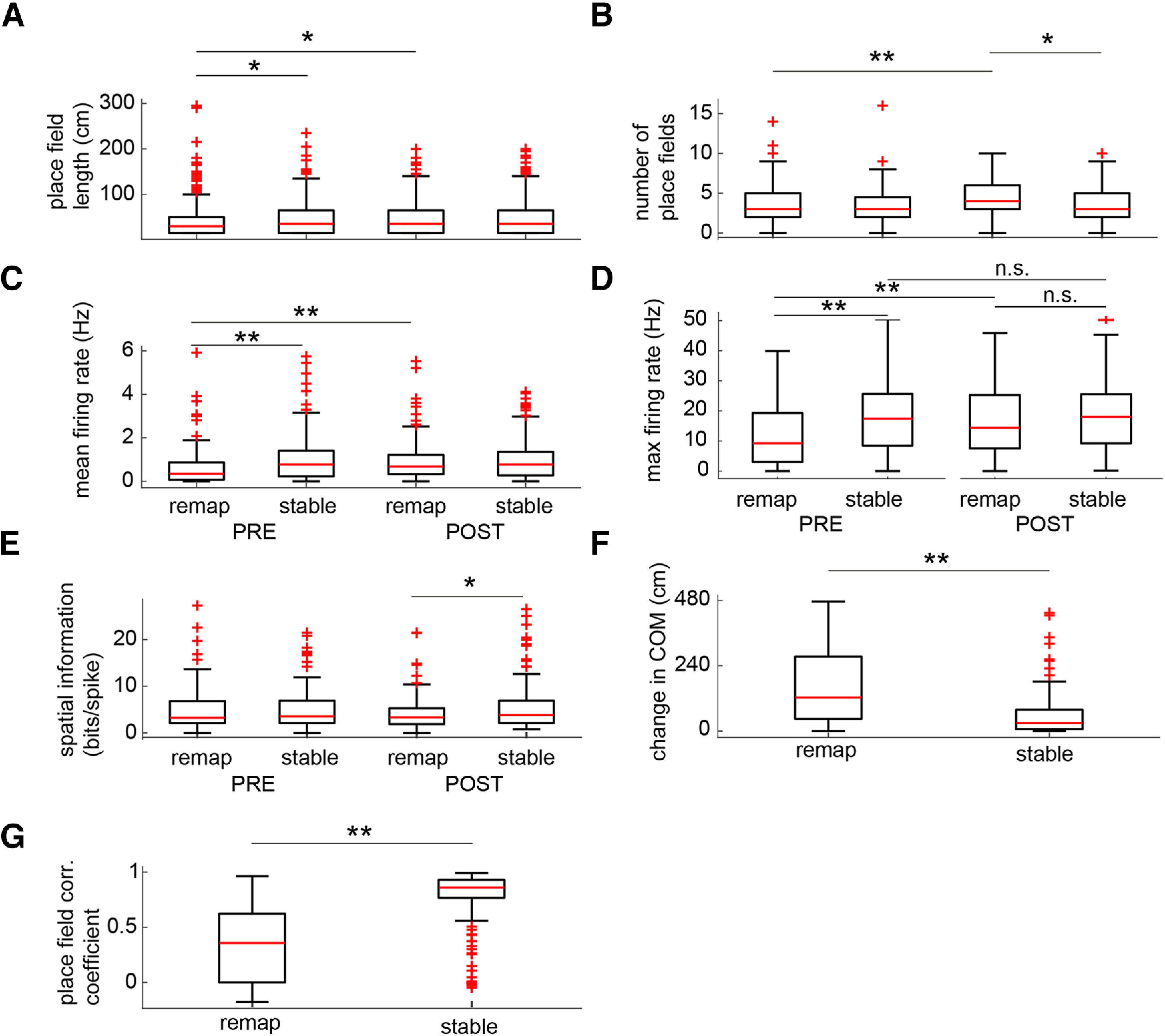
Place field properties of remapping and stable cells. ***A***, Remapping cells had smaller place fields before the contingency change, which grew to a size not significantly different from that of stable cells after learning. ***B***, The number of place fields expressed by remapping cells was not different from that of stable cells before learning, but increased after learning. ***C***, Mean firing rates were initially lower in remapping cells, but increased across learning to a level not significantly different from stable cells. ***D***, The same pattern observed in the mean firing rate data were observed in the maximal firing rate data. ***E***, Spatial information was greater in stable cells after learning compared with remapping cells, but neither group changed significantly across learning. ***F***, The centers of mass of remapping cell rate maps shifted significantly more than those of stable cells across learning. ***G***, Similarly, rate maps of remapping cells were significantly more decorrelated after learning than those of stable cells. *p < 0.05, **p < 0.001, n.s. indicates not significant.

**Figure 5.**
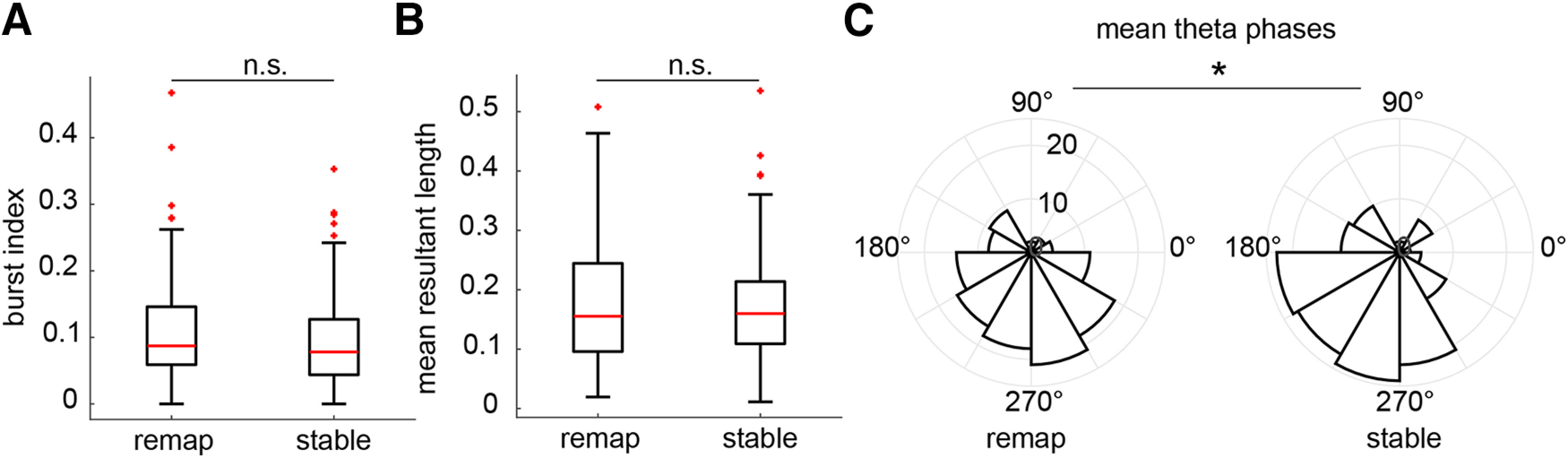
Bursting and theta modulation of remapping and stable cells. ***A***, Remapping cells did not have higher burst index scores than stable cells. ***B***, The degree of theta modulation, measured as the mean resultant length of the circular distribution of spike theta phases, was not different between remapping and stable cells. ***C***, However, the mean theta phases of remapping cells were shifted to later phases compared with those of stable cells. *p < 0.05, n.s. indicates not significant.

Investigating place field properties of remapping and stable cells in greater detail, we found that place field sizes were smaller in remapping cells before learning, and increased across learning to a size not different from stable cells (Wilcoxon rank-sum test: PRE: *z* = −2.72, *p* = 0.007; POST: *z* = −0.46, *p* = 0.065; Remapping: *z* = −3.02, *p* = 0.003; Stable: *z* = −0.35, *p* = 0.73; Fig. 4*A*). The number of fields expressed by both remapping and stable cells increased with learning, though to a larger extent than in remapping cells (Wilcoxon rank-sum test: PRE: *z* = 0.12, *p* = 0.090; POST: *z* = 3.21, *p* = 0.0013; Wilcoxon signed-rank test: Remapping: *z* = −4.25, *p* < 0.001; Stable: *z* = −2.16, *p* = 0.031; Fig. 4*B*). Mean and maximal firing rates in remapping cells were lower in remapping cells before learning, and then rose across learning to a level not different from those of stable cells (mean rates: Wilcoxon rank-sum test: PRE: *z* = −4.00, *p* < 0.001; POST: *z* = −0.23, *p* = 0.82; Wilcoxon signed-rank test: Remapping: *z* = −3.98, *p* < 0.001; Stable: *z* = 0.78, *p* = 0.44; maximal rates: Wilcoxon rank-sum test: PRE: *z* = −4.55, *p* < 0.001; POST: *z* = −1.65, *p* = 0.094; Remapping: *z* = 3.45, *p* < 0.001; Stable: *z* = 0.15, *p* = 0.88; Fig. 4*C*,*D*). Stable cells carried more spatial information than remapping cells after learning (Wilcoxon rank-sum test: PRE: *z* = −0.96, *p* = 0.34; POST: *z* = −2.13, *p* = 0.04; Wilcoxon signed-rank test: Remapping: *z* = 1.31, *p* = 0.19; Stable: *z* = 1.28, *p* = 0.20; Fig. 4*E*). Confirming the validity of cell identification procedure, we found that the spatial distribution of remapping cell firing changed significantly across learning compared with stable cells, measured both as change in the rate map center of mass (Wilcoxon rank-sum test: *z* = 6.81, *p* < 0.001; Fig. 4*F*) and reduced rate map correlations (Wilcoxon rank-sum test: *z* = −10.59, *p* < 0.001; Fig. 4*G*). Next, we investigated burst firing and coordination of firing by the theta rhythm. Remapping cells did not have significantly greater burst index scores than stable cells (Mizuseki and Buzsáki, 2013; Wilcoxon rank-sum test: *z* = 1.69, *p* = 0.091; Fig. 5*A*). The magnitude of theta modulation, as defined by the mean resultant length of the circular distribution of spike theta phases, was also not significantly different between remapping and stable cells (Wilcoxon rank-sum test: *z* = −0.11, *p* = 0.091; Fig. 5*B*). However, the mean theta phases of significantly modulated remapping cells (126 of 133 cells, 94.7%) were shifted to later phases compared with stable cells (138 of 144 cells, 95.8%; common median test: *z* = 4.92, *p* = 0.027; Fig. 5*C*). These findings reveal that in addition to being more sensitive to learning, remapping cells also exhibited important differences in their coding properties compared with stable cells.

### Remapping cells increase their replay participation during learning

**Figure 6.**
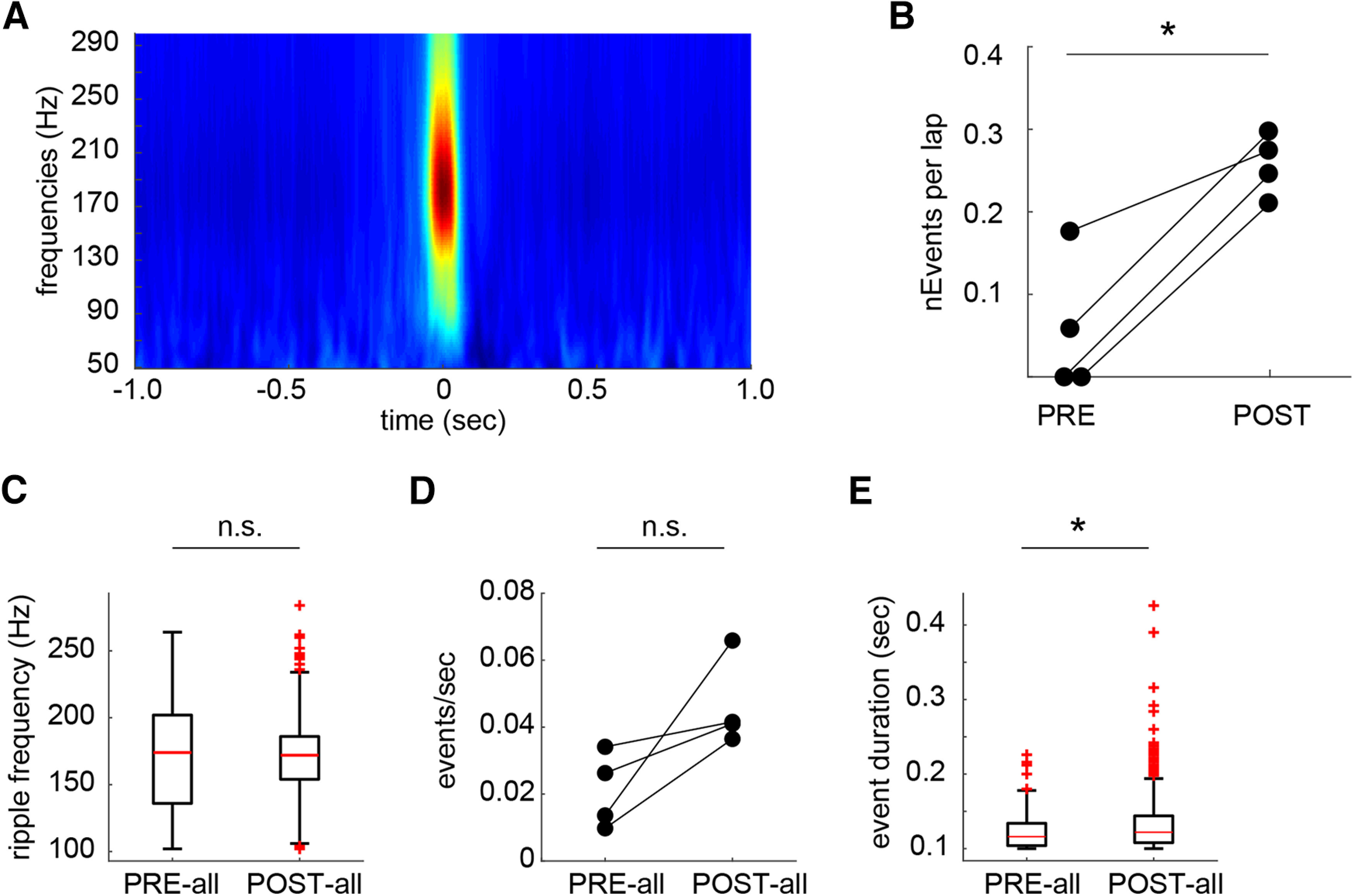
Ripple properties. ***A***, Local field potential recordings showed a large increase in power in the ripple band (100–250 Hz) during candidate replay events. ***B***, There was a significant increase in the frequency of replay events after the introduction of shock on the choice arms. ***C***, The oscillatory frequency of ripples was not significantly different across learning. ***D***, The rate of ripple generation was not significantly different across learning. ***E***, Ripples were significantly longer after learning. *p < 0.05, n.s. indicates not significant.

The main purpose of our study was to test whether awake replay of hippocampal place cell sequences, which has been proposed to function as a mechanism to stabilize plasticity following learning (Dupret et al., 2010; Carr et al., 2011), might serve such a function with regard to the partial remapping induced by aversive learning in the “Learning” sessions. We identified candidate replay events as brief increases in the population-wide firing rate coinciding with SWRs (Fig. 6*A*). Neither the ripple frequency of these events (Wilcoxon rank-sum test: *z* = 0.46, *p* = 0.65; Fig. 6*B*) nor their rate of generation (*t*_(3)_ = −2.57, *p* = 0.082; Fig. 6*C*) changed across learning, but their duration did increase (Wilcoxon rank-sum test: *z* = −2.46, *p* = 0.014; Fig. 6*D*), consistent with a recent report showing that ripple duration increases with learning and memory demands in rats (Fernández-Ruiz et al., 2019).

**Figure 7.**
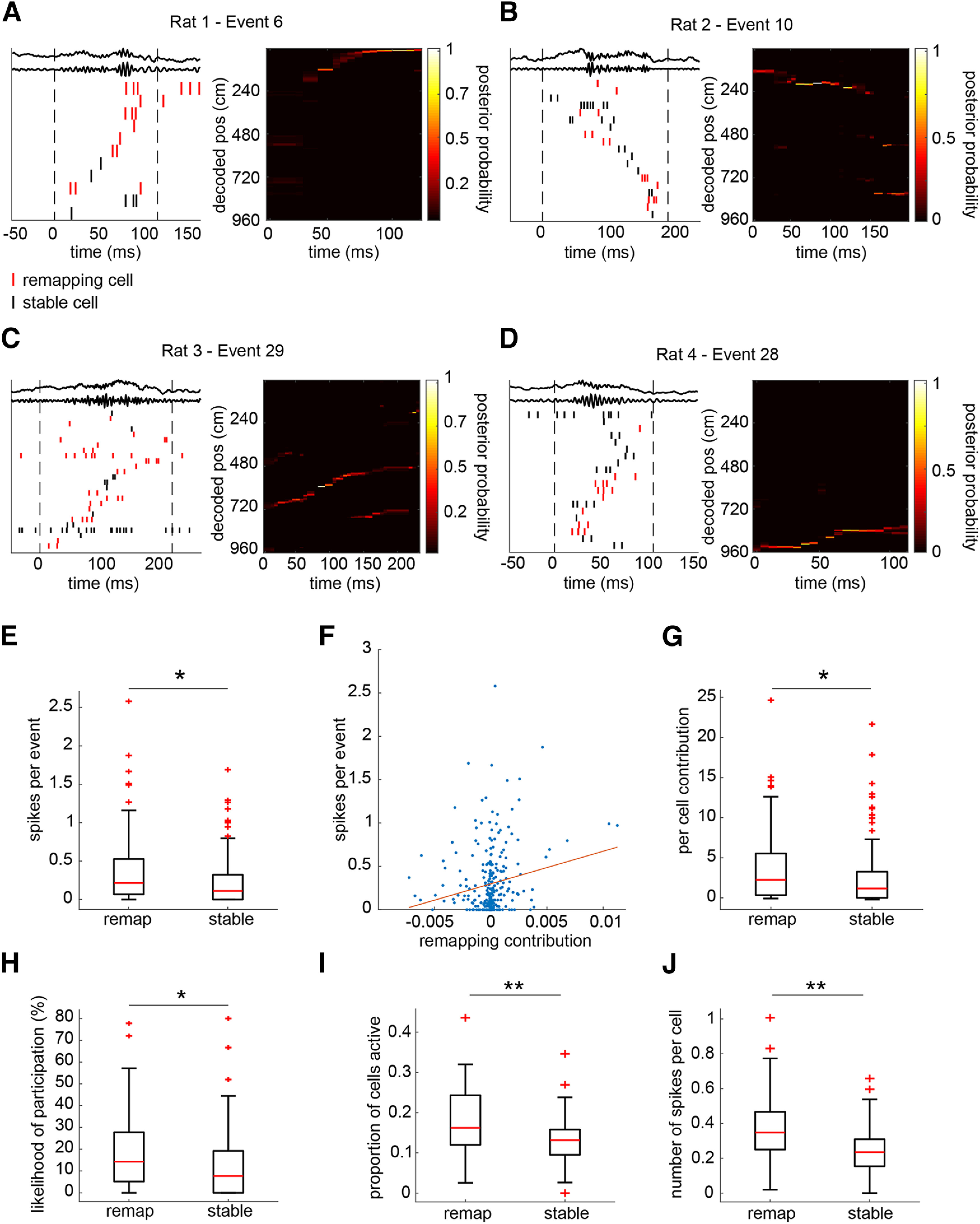
Remapping cells show greater involvement in replay. ***A*–*D***, Example replay events showing the raw and ripple band-filtered LFP and spikes color coded by cell identity (remapping, red; stable, black) and ordered according to their location of peak firing (left; vertical dashed lines show the temporal borders of the event) and heat maps showing the posterior probabilities calculated using Bayesian decoding (right). ***E***, Remapping cells fired more spikes per replay event than stable cells. ***F***, There was a significant correlation between remapping contribution and number of spikes fired per replay event in the combined remapping and stable population. ***G***, Remapping cells made a larger contribution to the correlation between elapsed time and decoded position within each replay event. ***H***, Remapping cells were more likely than stable cells to participate within a given replay event. ***I***, The proportion of the remapping cells population participating within any given event was larger than that of the stable population. ***J***, The number of remapping cell spikes, normalized by the number of remapping cells, fired within a given replay event was larger than the corresponding normalized number of stable cell spikes. *p < 0.05, **p < 0.001.

A candidate event was classified as a replay event if the correlation between decoded position and time within the event was greater than that of the 95th percentiles of each of two control distributions calculated from two separate shuffling procedures (see Materials and Methods). Replay events became more frequent after the contingency change (i.e., introduction of shock), indicating an important role for these events in learning (*t*_(3)_ = −5.71, *p* = 0.0107; Fig. 4*B*). The very small number of replay events observed before the contingency change (PRE: 0 events in rats 1 and 2, 1 event in rat 3, 3 events in rat 4) precluded any further examination of replay before learning (these events were excluded from further analysis). In total, there were 98 significant replay events during the POST-all epoch, which consisted of all trials occurring after the contingency change (18, 27, 28, and 25 events in rats 1–4, respectively; Fig. 7*A*–*D*).

We asked whether there was a difference between remapping and stable cells in terms of their participation within these replay events. Consistent with our hypothesis, we found that remapping cells fired significantly more spikes during replay events than stable cells (Wilcoxon rank-sum test: *z* = 3.03, *p* = 0.0024; Fig. 7*E*). Supporting this, we found a significant correlation between remapping contribution and the number of spikes fired during replay across the place cell population (*n* = 277 cells; Pearson correlation coefficient = 0.1879, *p* = 0.0017; Fig. 7*F*). To measure the contribution that individual cells made to the correlations between time and decoded position, which is the hallmark of hippocampal replay, we used a previously published cell-specific shuffling technique (Grosmark and Buzsáki, 2016). This analysis showed that remapping cells made significantly greater contributions than stable cells to the time–position correlation (Wilcoxon rank-sum test: *z* = 2.88, *p* = 0.0040; Fig. 7*G*). These results were not skewed by remapping cells firing a large number of spikes in a minority of replay events, as we found that remapping cells were more likely to fire at least one spike within any given event (Wilcoxon rank-sum test: *z* = 3.23, *p* = 0.0012; Fig. 7*H*). Further, examining individual events rather than cells confirmed that events contained a greater proportion of the remapping cell population (Wilcoxon signed-rank test: *z* = 4.87, *p* < 0.001; Fig. 7*I*) as well as a greater number of remapping cell spikes compared with stable cells (Wilcoxon signed-rank test: *z* = 4.97, *p* < 0.001; Fig. 7*J*).

**Figure 8.**
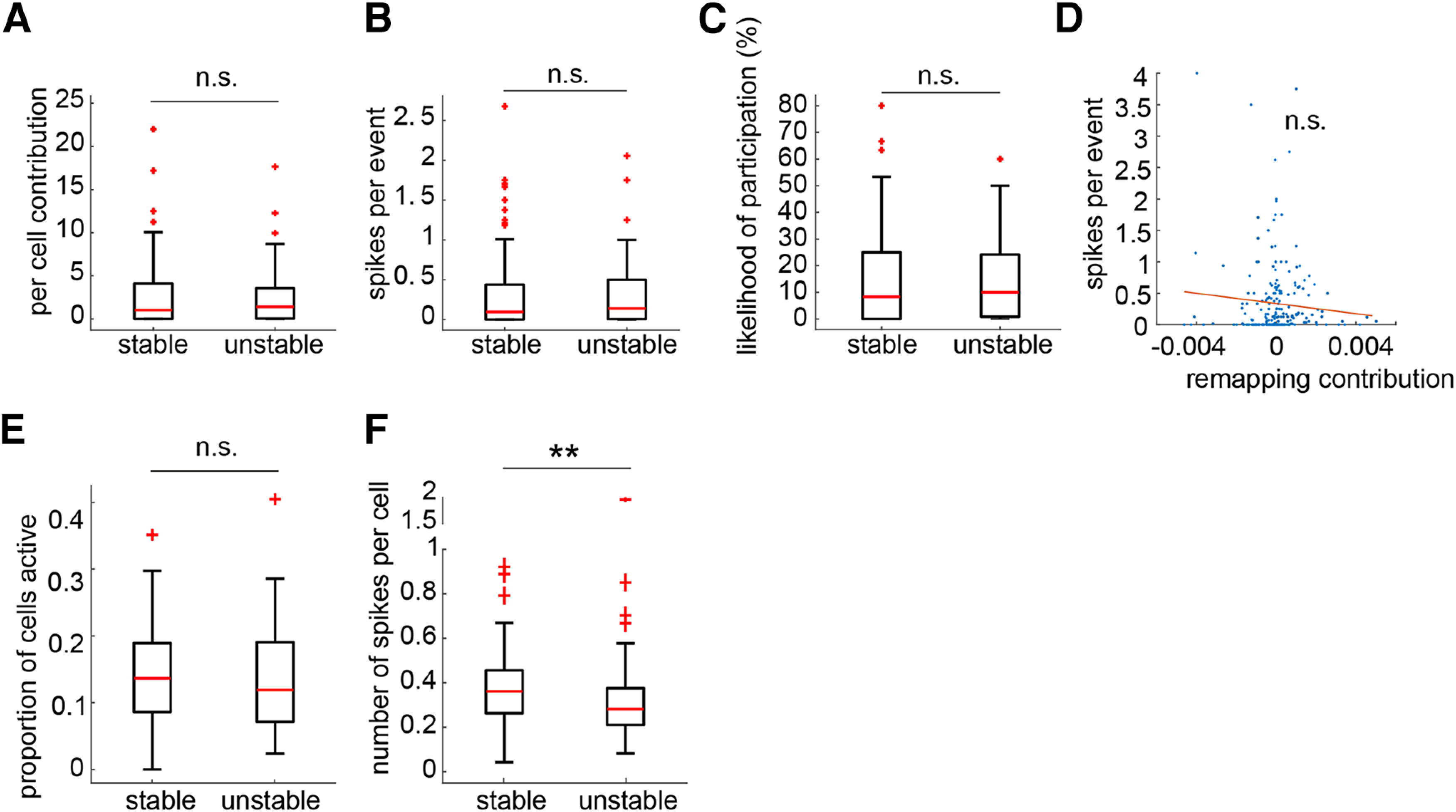
Unstable cells do not show greater involvement in replay during CTRL sessions. ***A***, Stable and unstable cells made similar contributions to the correlation between elapsed time and decoded position within replay events. ***B***, Stable and unstable cells fired similar numbers of spikes during replay events. ***C***, Stable and unstable cells had similar likelihoods to fire spikes within replay events. ***D***, There was no correlation between the remapping contribution of a cell and the number of spikes it fired during replay. ***E***, The proportions of the stable and unstable populations active within replay events were not significantly different. ***F***, The number of spikes per stable cell within replay events was not significantly different from the number of spikes per unstable cell. **p < 0.001, n.s. indicates not significant.

To confirm that these differences were because of learning, we turned to the control sessions. Because all place fields will undergo a certain amount of change or fluctuation (Mankin et al., 2012) over time, which, like any biological process, will vary to some degree across units, we were able to split the control session place cell population using the same cell identification procedure into stable and unstable populations, with the unstable population being equivalent to the remapping population in the Learning sessions (*n* = 123 stable cells, *n* = 105 remapping cells). There were no significant differences between these two groups in terms of per cell contribution, spikes per event, or likelihood of participation (Wilcoxon rank-sum test: *z* = −1.60, −1.67, and −1.48; *p* = 0.11, 0.10, and 0.14, respectively; Fig. 8*A–C*). Further, the correlation between remapping contribution and spike count was abolished in the CTRL sessions (Pearson correlation coefficient = 0.085, *p* = 0.203; Fig. 8*D*). Repeating our analysis of individual replay events, we found no difference in the proportions of more and less stable cell populations active within the events (Wilcoxon signed-rank test: *z* = −1.21, *p* = 0.227; Fig. 8*E*), but we did find that the normalized number of stable cells spikes was significantly greater than for unstable cells (Wilcoxon signed-rank test: *z* = −2.35, *p* = 0.019; Fig. 8*F*).

**Figure 9.**
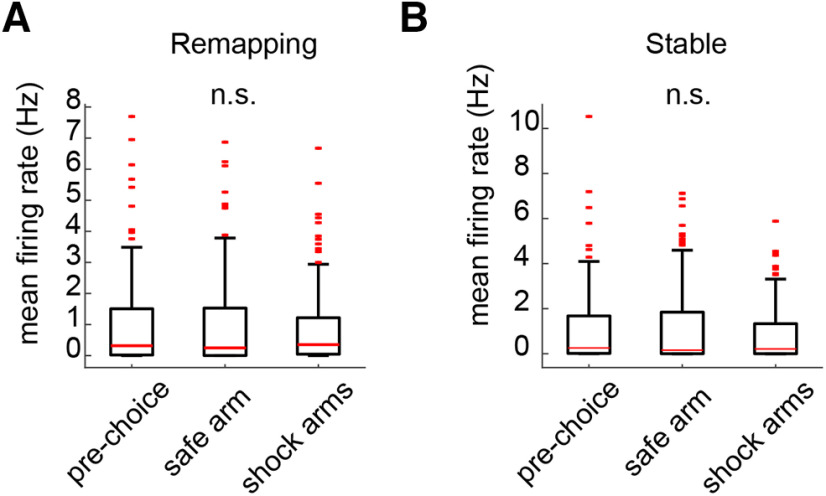
The firing of remapping and stable cells is evenly distributed across the maze. ***A***, Mean firing rates of remapping cells were not different across the 3 different maze arm types. ***B***, Similarly, there was no difference in firing across the 3 arm types in stable cells. n.s. indicates not significant.

Because animals ran more trials on the safe arm than on the shock arms (55.9% trials on the safe arm postcontingency change), we wondered whether differences in remapping and stable cell distribution across the three arm types (prechoice, safe, and shock) could interact with an experience-dependent effect on replay (e.g., more replay of the safe arm because of more safe arm experience) to produce the enhancement of replay activity in remapping cells. However, we did not observe any differences in mean firing rates across the three arm types for either remapping or stable cells (Kruskal–Wallis test: remapping cells: *H*_(398)_ = 0.093, *p* = 0.95; stable cells: *H*_(431)_ = 1.23, *p* = 0.54; Fig. 9*A*,*B*), suggesting that any experience-dependent effect on replay would affect both cell populations equally.

### Replay events may not represent planning

**Figure 10.**
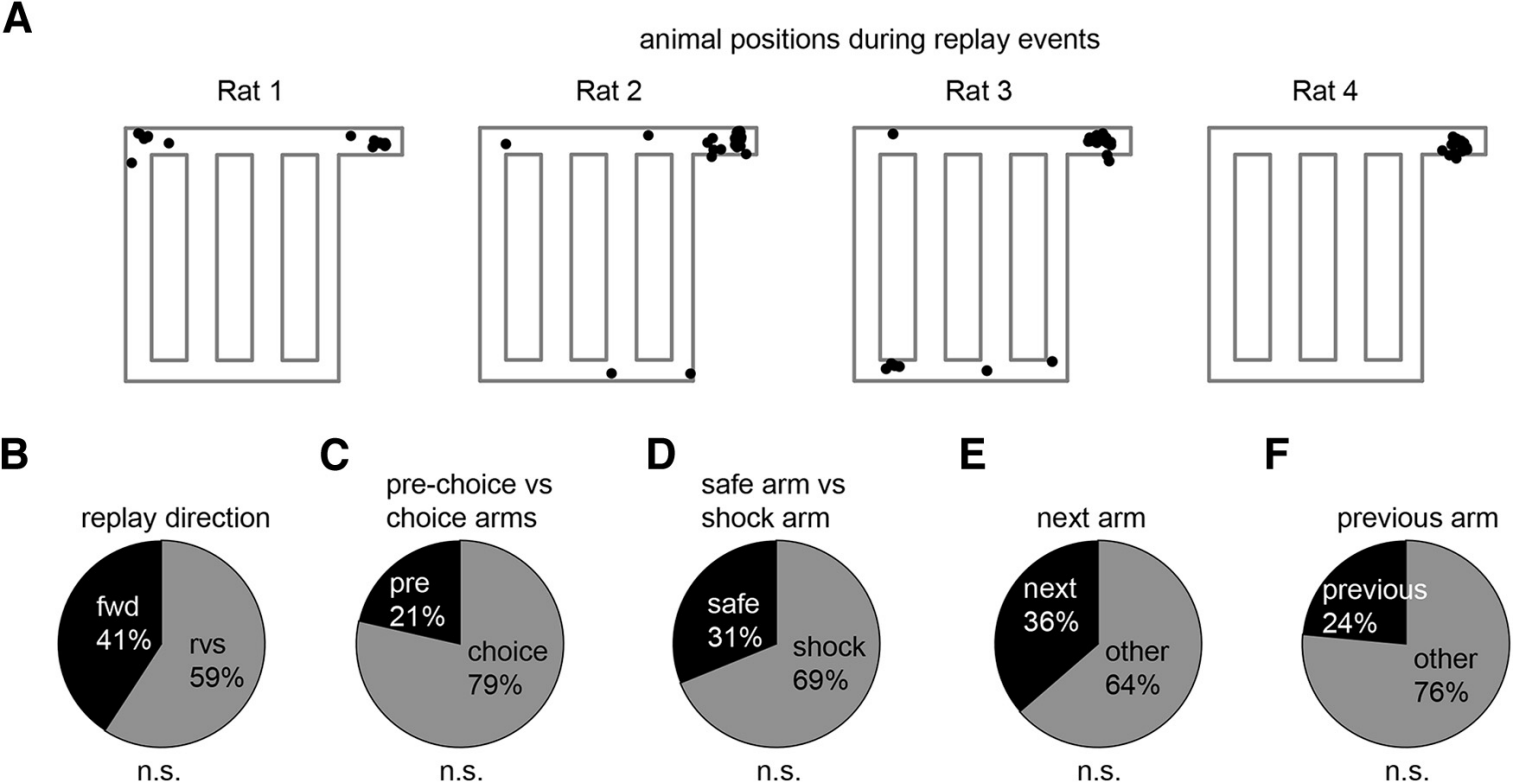
Replay events are unlikely to underlie route planning. ***A***, Black dots represent the maze locations at which replay events occurred. Most replay events occurred at the large reward location where the animal remained confined for 10 s after each trial. A smaller number were also observed at the small reward location. A very small number was observed at the choice points, but only in rats 2 and 3. ***B*–*F***, No bias was observed for replay events to encode either forward or reverse trajectories (***B***), the prechoice or the choice arms (***C***), the safe or the shock arms (***D***), the next traversed choice arm (***E***), or the previously traversed arm (***F***). n.s. indicates not significant.

We plotted the maze positions at which each replay event occurred. The vast majority occurred at the main reward site in the interval between successive trials (Fig. 10*A*). In three of the four animals, there were a small number that occurred at the small reward location. In two of the animals, there were a small number of events immediately before entry into the choice arms; in rat 2, both events were decoded as forward replays up a shock arm, but the animal ultimately chose the safe arm; in rat 3, all six events occurred during forced-choice trials after the animal had learned the identity of the safe and shock arms, and four of these consisted of two pairs of replays, with the first representing a trajectory up a shock arm followed by a second up the safe arm, suggesting the possibility that they were involved in deliberation.

We examined the content of the individual replay events more closely in an effort to determine whether there was any further evidence that the events were guiding behavior. Forward replay events (i.e., in which the decoded position progresses in a forward direction around the maze) occurred with a frequency that was not different from reverse replay, arguing against an exclusive role in planning (binomial test: chance level = 50%, *p* = 0.86; Fig. 10*B*). We did not observe any bias of replay for paths along the choice arms compared with paths that only included the prechoice portion of the maze (i.e., the path from the top right to bottom left; chance level of encoding prechoice arm = 25%, *p* = 0.48; Figs. 1, 10*C*), any bias toward paths along safe arm (chance level of encoding safe arm = 33.3%, *p* = 0.72; Fig. 10*D*), or paths along the next arm (chance level = 33.3%, *p* = 0.82; Fig. 10*E*; this analysis omitted forced-choice trials), supporting our interpretation that replay did not appear to support route planning in this particular behavioral paradigm, and adding further evidence that the greater number of trajectories on the safe arm did not skew the content of replay. Finally, we found no bias toward replay of the previous choice arm (chance level, 33%; binomial test, *p* = 0.180; Fig. 10*F*; this analysis also omitted forced-choice trials), suggesting that replay was not reinforcing the spatial representation of the previous choice. Thus, the content of replay seems to correspond to trajectories distributed around the entire maze, and these trajectories appear to be selected randomly.

Together, our analyses of replay events during aversive learning suggest that the role of replay may be in facilitating the storage or stabilization of a new spatial memory. However, given the relatively small number of replay events and the possibility that not all events necessarily serve the same purpose, a role in planning for individual replay events cannot be ruled out.

### Firing properties during sharp-wave ripples

**Figure 11.**
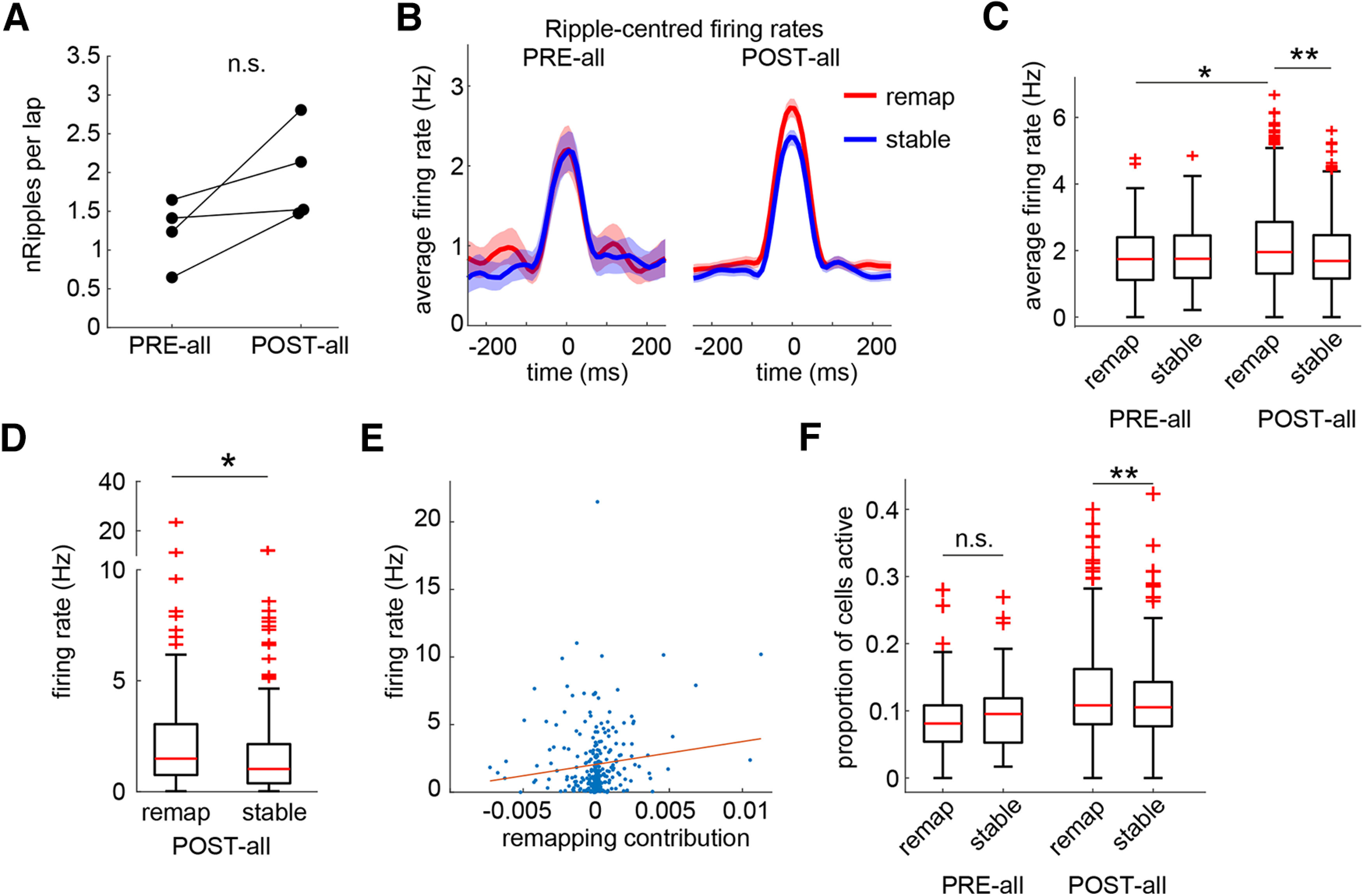
Remapping cells have higher firing rates during non-replay-associated ripples. ***A***, There was no significant difference in the rate of ripple generation after learning. ***B***, The average ripple-centered remapping cell firing rate increased after learning. Solid line shows the median values calculated across all ripples; shaded area is the 95% confidence interval calculated using bootstrap sampling. ***C***, This increased average rate was significantly higher than that of stable cells. ***D***, Similarly, the ripple-centered firing rates of individual remapping cells were higher after learning than those of stable cells. ***E***, There was a significant correlation between the remapping contributions and ripple-centered firing rates in the pooled remapping and stable cell population. ***F***, The proportion of the remapping cell population active within a given ripple was higher after learning than that of the stable cell population. *p < 0.05, **p < 0.001, n.s. indicates not significant.

**Figure 12.**
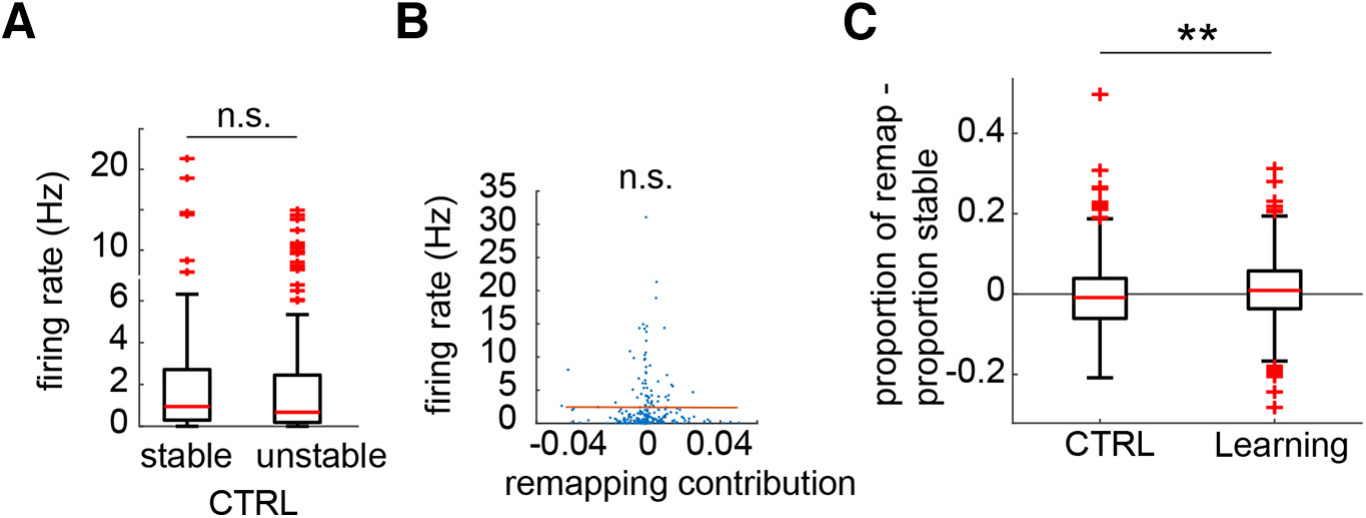
Ripple-centered firing of stable and unstable cells during CTRL sessions. ***A***, Ripple-centered firing rates of individual unstable cells were no higher than those of stable cells during POST-all in CTRL sessions. ***B***, Unlike in the Learning sessions, there was no correlation between remapping contribution and ripple-centered firing rate in the pooled stable and unstable cell populations. ***C***, Learning session ripples contain a greater proportion of the remapping population relative to the stable population, while the opposite is observed in CTRL sessions. **p < 0.001, n.s. indicates not significant.

We wondered whether a general upregulation of firing during SWRs might underlie the increased participation of remapping cells in replay events. We analyzed all SWRs that did not co-occur with statistically significant replay events from both the precontingency and postcontingency change periods (this analysis used the PRE-all epoch, consisting of PRE with the addition of five preceding free-choice trials, see Methods and Materials; PRE-all, *n* = 84; POST-all, *n* = 814). The rate of ripple occurrence did not increase significantly across learning (Fig. 11*A*). Calculating the average remapping cell and stable cell ripple-centered firing rates within each ripple, we found that remapping cells increased their firing rates across learning (Wilcoxon rank-sum test: remapping: *z* = −2.79, *p* = 0.005; stable: *z* = −0.88, *p* = 0.38) to a level that was significantly higher than that of stable cells (Wilcoxon signed-rank test: PRE-all: *z* = 0.24, *p* = 0.81; POST-all: *z* = 5.02, *p* < 0.001; Fig. 11*B*,*C*). Similar to replay, we found a significant correlation between remapping contribution and ripple firing rate for individual place cells after the contingency change (remapping and stable cells: Wilcoxon rank-sum test: *z* = 2.60, *p* = 0.009; pooled population: Pearson correlation coefficient = 0.132, *p* = 0.028; Fig. 11*D*,*E*); in CTRL sessions, this correlation was abolished (stable vs unstable cells: Wilcoxon rank-sum test: *z* = 0.98, *p* = 0.33; Pearson correlation coefficient = −0.0031, *p* = 0.963; Fig. 12*A*,*B*). The proportions of the remapping and stable populations active within any given event increased across learning, but this increase was significantly larger in remapping cells (Remapping: Wilcoxon rank-sum test: *z* = −4.58, *p* < 0.001; Stable: Wilcoxon rank-sum test: *z* = −2.45, *p* = 0.014; PRE-all: Wilcoxon signed-rank test: *z* = −0.17, *p* = 0.86; POST-all: Wilcoxon signed-rank test: *z* = 5.16, *p* < 0.001; Fig. 11*F*); this difference was reversed in CTRL sessions (Wilcoxon signed-rank test: *z* = −2.79, *p* = 0.005; difference from remapping: Wilcoxon rank-sum test: *z* = −5.27, *p* < 0.001; Fig. 12*C*).

## Discussion

Our data confirm previous findings that aversive learning in a familiar environment induces partial remapping (Wang et al., 2012; Wu et al., 2017). We show for the first time that the subset of cells that remap express an enhanced recruitment into awake SWRs and replay events compared with nonremapping stable cells. This enhanced recruitment of remapping cells cannot be explained by increases in in-field firing rates; though the firing rates of remapping cells did increase across learning, they did not surpass those of stable cells. Further, because remapping cell firing was distributed along all arms of the maze, differences in behavior at specific locations on the maze, such as increased latency to enter shock arms and greater choice preference for the safe arm after learning are also unlikely to explain this enhancement. Thus, our data uncover a link between the excitability of a cell, specifically during SWRs, and the reorganization of its spatial pattern of firing during locomotion, when theta oscillations dominate the local field potential.

While we have found it useful to classify cells as belonging to either remapping or stable place cell populations, we point out here that they may instead exist on a continuum with highly remapping and highly stable cells at the extremes, a possibility highlighted by our correlational analyses (Figs. 7*F*, 11*E*).

### Replay as a memory storage mechanism occurring in remapping cells

Previous studies have shown that in reward learning, cells whose fields become clustered around the reward zones increase their firing during SWRs (Dupret et al., 2010). However, because reward increases firing rates during SWRs, and SWRs tend to involve cells that have place fields at the reward zone (Singer and Frank, 2009), it was not clear whether the enhanced contributions of reward zone cells was because of a unique role for these cells in replay, or simply a by-product of their place field locations. Our task, in which remapping and nonremapping cell place fields were both distributed along the track arms, circumvented this limitation, and our data show that cells involved in memory encoding increase their contributions during learning more than nonremapping stable cells. Thus, in our task, the location of the place field of a cell does not seem to be the primary determinant of its involvement in either SWRs or replay events.

Research into replay has focused on two roles it may play. First, there is substantial evidence for a role in trajectory planning. In typical goal-directed tasks, the trajectory, or trajectories, leading to the goal reward are thought to be replayed preferentially as a means of the animal planning and previewing where it must navigate to reach the goal (Pfeiffer and Foster, 2013; Widloski and Foster, 2022). Conversely, in a spatial paradigm featuring an aversive shock zone, but no goal zone, replay was shown to preferentially activate cells with fields in the shock zone (i.e., trajectories to be avoided; Wu et al., 2017). Similarly, replay has also been shown to encode a nonpreferred/unrewarded option when another rewarded option is available, indicating that replay can encode trajectories to avoid even in the absence of aversive stimuli (Gupta et al., 2010; Carey et al., 2019). Because replay has been reported both to encode trajectories to navigate, as well as those to avoid, it is impossible to make a prediction of replay content in our paradigm if replay were indeed to serve a role in planning; in our task, there is a part of the maze that is aversive and should be avoided, as well as safe arms that are preferentially navigated by the animal, and the studies cited above suggest that both should be replayed. Therefore, we cannot rule out a role for replay in trajectory planning in our task.

The second, not necessarily mutually exclusive, view of replay is that it underlies memory storage. Disruption of ripples during learning (Jadhav et al., 2012) and post-training periods (Girardeau et al., 2009) has been shown to impair rats trained on a hippocampus-dependent spatial memory task. Further, an increase in SWR incidence has been reported at novel goal locations, and the frequency of these events predicted task performance (Dupret et al., 2010). In a task designed to promote replay before memory-based choice, replay content was found to be decoupled from subsequent choice (Gillespie et al., 2021). Reverse replay, in particular, has been suggested to play an important role in memory encoding, as it is enhanced in novel environments and has been postulated to allow the linking of a reward with the sequence of places through which the animal had to travel to receive it (Foster and Wilson, 2006). Our data appear more consistent with this second view. First, we observed very few replay events at the choice points; instead, most events occurred at the large reward location, where the animal was confined for 10 s between trials. While this is not surprising, given that cessation of locomotion is necessary for brain state to transition from theta into slow-wave activity (Buzsáki, 1986), a prerequisite for SWRs, we would nevertheless expect a greater number of replays at the choice points if they were necessary for route planning/decision-making, as animals were free to stop at the choice points to deliberate over their options. Second, we did not observe any difference in the likelihood of involvement of the relatively neutral prechoice arms and the non-neutral (i.e., aversive or safe) choice arms. Third, we did not observe any preference of forward replay for the next traversed arm. Last, we did not find that forward replay occurred more frequently and, in fact, saw a trend, though not significant, toward a greater incidence of reverse replay. Together, these data indicate that replay during our task may serve to incorporate the spatial coding of the remapping cells into the larger hippocampal representation.

### Ripples versus replay

Ripples are brief (duration, 50–100 ms), high-frequency (150–300 Hz) network oscillations within the hippocampus that emerge during nonexploratory states such as slow-wave sleep, quiet rest, grooming, and eating/drinking (Buzsáki, 1986, 2015), and disruption in their expression results in significant memory impairments (Girardeau et al., 2009; Nakashiba et al., 2009; Ego-Stengel and Wilson, 2010; Jadhav et al., 2012; Wang et al., 2015). Typically, only a subset of ripples is found to contain significant replay of place field sequences (Foster and Wilson, 2006; Tingley and Peyrache, 2020). This raises the question of whether replay events and nonreplay ripple events serve different functions. Our finding that remapping cells had enhanced participation in both kinds of events suggests that both serve the same underlying function in our task, namely, to consolidate the incorporation of emotional and contextual information into the spatial representation of the maze. Further, recent work suggests that the heuristic approaches typically used in the field to identify replay events only identify the most salient trajectories, and more sophisticated techniques suggest that most ripple events do contain replay (Krause and Drugowitsch, 2022); this suggests that our ripple results may be best viewed as a replication of our replay results. Together, these data suggest the existence of a mechanism by which remapping cell excitability is enhanced during SWRs, such as a differential response relative to stable cells to the decreased activity of cholinergic and septal GABAergic inputs when transitioning from theta (Buzsáki, 2002).

### Conclusion

Our identification and examination of remapping cells show that increased coordination during ripples and replay events in these cells may facilitate the generation and stabilization of plasticity in the memory-encoding place cell network, thereby linking a spatially restricted experience to a representation of the larger environment in which it occurred. The enhancement of replay contribution in remapping cells suggests the existence of a mechanism to select those cells with the emergent coding properties that best support adaptive behavior for participation in replay. That these processes occur in a specific cell population, while a separate population of place cells exhibit stable spatial coding, demonstrates a mechanism whereby the hippocampus can both maintain a stable spatial representation of the environment while incorporating changing features of experiences that occur within that environment.
